# Fixation drift increases as a function of time-on-task in a brief saccade tracking study

**DOI:** 10.1371/journal.pone.0310619

**Published:** 2025-06-05

**Authors:** Lee Friedman, Oleg V. Komogortsev

**Affiliations:** Department of Computer Science, Texas State University, San Marcos, Texas, United States of America; University of Pecs Medical School, HUNGARY

## Abstract

Ocular fixations contain microsaccades, drift and tremor. We report an increase in the slope of linear fixation drift as a function of time-on-task (TOT). We employed a large dataset (322 subjects, multiple visits per subject). Subjects performed a random saccade task. The task, in which the target dot jumped randomly every one sec, was 100 sec in duration. For each fixation, we regressed eye position against time across multiple segment lengths (50, 100, 300, and 500 ms). We started with the first sample and continued until no further regressions were possible based on the segment length being evaluated. For each segment length, each fixation was characterized by a single value: the maximum slope over the segment length. The slopes were expressed in deg/sec. We were not interested in the direction of the linear drift, so we took the absolute value of the slope as a measure. For data analysis, each 100 sec task was divided into five 20 sec epochs. In an attempt to partially replicate an earlier study of fixation drift over time [1], we also measured drift as the mean velocity of an eye-position signal that had been low-pass filtered at 30 Hz. We found that both methods detected significant drift over our task. For each visit, subjects were tested in two sessions, approximately 20 min apart. Generally, we found statistically significant session effects indicating that drift started at a higher level and increased at a higher rate over the second session. We report that the mean velocity method detects distinctive types of drifting fixation trajectories including curvilinear drift and irregular oscillation. Our findings extend the observations of increased drift over time by [1] to other measures of drift and to a much shorter time interval (100 sec vs two hrs) and a simpler task.

## Introduction

It is well established that during “ocular fixation” the eye is not still. There are three types of eye-movements that occur: microsaccades, drift and tremor [2]. DiStasi *et al*. [1] is the first, and as far as we are aware, only publication to report that fixation drift increases as a function of fatigue. In that study, subjects performed a simplified air traffic control task. The entire task took two hours to complete and consisted of four 30-minute blocks. Drift periods were defined as the eye-position epochs between microsaccades, saccades, overshoots and blinks. Drifts shorter than 200 ms were discarded. For each drift, the key measures were mean and peak velocity. These authors noted a statistically significant linearly increasing drift mean velocity and peak velocity from the first 30 min block to the last 30 min block. The authors conclude that increased fixation instability occurs with time on task (TOT).

Although that prior study [1] stimulated our interest in this topic, our study is distinctive in a number of important ways: (1) Di Stasi *et al*. [1] studied the effect of TOT on drift velocity over a two-hour period whereas our study evaluates changes in drift related to TOT where the entire task was completed in 100 seconds. (2) Di Stasi *et al*. [1] evaluated drift during the performance of a simplified air traffic control task. We evaluated drift during a simple, 100 sec random saccade tracking task. (3) The [1] study was based on the evaluation of twelve subjects whereas our study included data from 322 subjects. Since subjects were evaluated in two sessions over multiple rounds, our results were based on 1,125 separate eye movement recordings. Despite these clear differences between the Di Stasi *et al*. [1] study and our study, both found evidence for increased fixation drift as a function of TOT.

DiStasi *et al*. [1] detected drift as a function of mean velocity of low-pass filtered position signals. In our view, it is not intuitively obvious what sort of fixation trajectories would be classified as drift with this method. Therefore, in the present study, we attempt to illustrate the type of fixation trajectories detected by this method.

## Materials and methods

### The eye tracking database

The eye tracking database employed in this study is fully described in [3] and is labeled “GazeBase” It is publicly available (https://figshare.com/articles/dataset/GazeBase_Data_Repository/12912257). The data were accessed on April 12, 2024. The authors had no access to information that could identify individual participants during or after data collection.

All details regarding the overall design of the study, subject recruitment, tasks and stimuli descriptions, calibration efforts, and eye tracking equipment are presented there. There were nine temporally distinct “rounds” over a period of 37 months, and round one had the largest sample. We only analyzed rounds one, two and three with the largest subject numbers (322, 136, and 105, respectively). For round one, there were 151 females and 171 males. Subjects in each successive round needed to have been present in all preceding rounds. Briefly, subjects were initially recruited from the undergraduate student population at Texas State University through email and targeted in-class announcements. Each session consisted of seven tasks. There were two sessions per visit, approximately 20 min apart. The only task employed in the present study was the random saccade task. During the random saccade task, subjects were to follow a white target on a dark screen as the target was displaced at random locations across the display monitor, ranging from ± 15 and ± 9 of degrees of visual angle (dva) in the horizontal and vertical directions, respectively. The minimum amplitude between adjacent target displacements was two dva. At each target location, the target was stationary for one sec. There were 100 target positions per task (100,000 samples per task). The target positions were randomized for each recording. The distribution of target locations was chosen to ensure uniform coverage across the display. Monocular (left) eye movements were captured at a 1,000 Hz sampling rate using an EyeLink 1000 eye tracker (SR Research, Ottawa, Ontario, Canada).

The gaze position signals for the random saccade task were classified into fixations, saccades, post-saccadic oscillations (PSOs) and various forms of artifact, using an updated version of our previously reported eye-movement classification method [4]. Although the code for actual saccade detection was complex, we describe it here in a very brief format. Saccade detection was based on an analysis of smoothed radial velocity. To detect saccades, the first step was to look for an event with a radial velocity above 100 deg/sec. The start and end of the saccade were based local velocity minima preceding and following the peak radial velocity of the event.

### Screening fixations

#### Removing fixations adjacent to artifact.

Any fixation which was immediately preceded or followed by any type of artifact (e.g., blink artifact), was excluded from this study.

#### Removal of fixations that are part of Square Wave Jerks.

According to [5], one type of saccadic intrusion, “Square Wave Jerks” (SWJ) are:

“...small (typically 0.5 degree), horizontal, involuntary saccades that take the eyes off the target and are followed, after an intersaccadic interval of about 250 milliseconds, by a corrective saccade that brings the eyes back to the target. They may occur in normal individuals at frequencies of 20 per minute or greater." Page 250.

Since fixations during these SWJ are fundamentally different from other fixation types, we wanted to exclude them. To this end, we develop a MATLAB (Natick, Massachusetts) script to detect SWJ and remove the fixations associated with SWJ from our dataset. To illustrate the results, for round one only, we found a total of 1,467 SWJ. Of 322 subjects, 54 had no SWJs. Of the remaining 268 subjects, 55 had only one SWJ. The median number of SWJ per subject was 2.5 (25th percentile = one, 75th percentile= five SWJ per subject). One subject had 26 SWJ in session one and 22 SWJ in session two. Our MATLAB script for detecting SWJ, 61 eye movement datasets, and 89 example images of SWJ are available online at https://hdl.handle.net/10877/18499.

### Calculation of fixation linear drift for each included fixation

**Fig 1:**
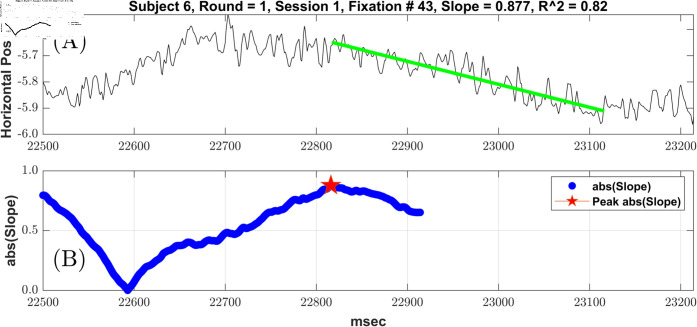
Illustration of the fixation drift evaluation method. (A) An example fixation from subject six, round one, session one. This was fixation number 43. It was 715 ms in length. The horizontal position is displayed. (B) This is a plot of 416 slopes. Starting at the first point, we regressed the first 300 eye-position data points onto a vector of ms numbers from 1:300. The slope of this regression represents the slope of eye position over this interval. Since we were interested in quantifying drift regardless of direction we take the absolute value of the slope. The raw slope expresses the drift in degrees per ms, so we multiply the slope by 1000 to obtain the slope per second. The red star is the point with the highest slope (absolute value). The green line in (A) is the fitted regression line plotted on the horizontal position signal starting at the point of maximum slope.

As illustrated in Fig 1(A), we started with the position data (either horizontal or vertical). This particular fixation is 715 ms long. In the illustrated case, we were fitting a line that was 300 ms in length. (We also tested, and will report on, other lengths (50, 100, or 500 ms)). For this particular analysis, the fixation has to be at least 301 ms in duration. Shorter fixations were not analyzed. The first step was to regress the position signal, starting at sample one of each fixation, against a vector from one to 300 in steps of one. Because we were interested in quantifying drift regardless of direction, we take the absolute value of the slope. The raw slope was expressed in degrees per ms, so we multiply the slope by 1000 to get degrees per second. Once we have the slope starting at point one, we then proceed to the next sample and repeat the analysis. This keeps going until the last sample that allows for a test of a 300 ms linear fit. In Fig 1(B), we plot the 416 slopes for this fixation. The measurement of drift was taken as the largest such slope for each fixation.

### Calculation of mean velocity for each included fixation

In addition to linear drift, we wanted to measure drift in the same manner as [1]. In [1], fixation position signals were low pass filtered (Butterworth, 30 Hz, order = 13). We assume that the filter employed by [1] was not zero-phase since they do not mention this. Butterworth filters inherently introduce a non-linear phase shift, especially near the cutoff frequency, which can be a delay or a phase shift depending on the filter’s order and the frequency of the signal. We used a 5th order Butterworth low-pass filter in a zero-phase approach (which effectively doubles the order of the filter). Our zero-phase filter introduces no phase delay or phase shift. Our signals were collected with a sampling rate of 1000 Hz whereas the data in [1] were sampled at 500 Hz. It is not clear how [1] computed velocity. We used the first derivative of a Savitzky-Golay filter (order = two, window length = seven ms) to compute velocity. In addition to horizontal and vertical mean velocity, we also calculated mean radial velocity using the low pass filtered position signals:

$$Radial\;Velocity=\sqrt{HorizontalVel^{2}+VerticalVel^{2}}$$ (1)

**Fig 2:**
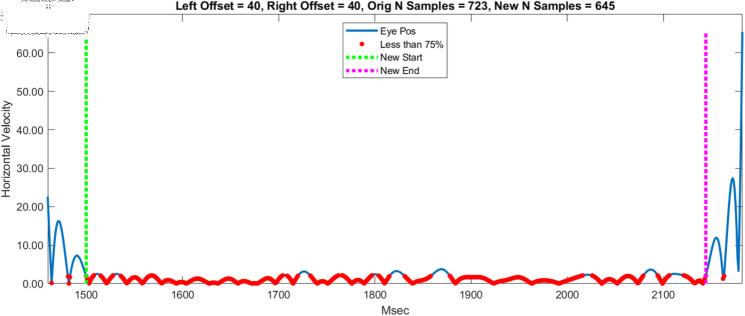
This figure illustrates our adaptive trimming method to select parts of a velocity signal that were not affected by signal transients introduced by the low pass filter. The blue line is the untrimmed velocity signal. The red points were all < =  the 75th percentile of the entire velocity signal. The green vertical line was the first sample (from the left) of four consecutive samples that were < =  the 75th percentile. This was taken as the new starting sample of the fixation. The magenta vertical line was the first sample (from the right) of four consecutive samples that were < =  the 75th percentile. This was taken as the new ending sample of the fixation.

DiStasi *et al*. [1] “...removed 10 ms from the start and end of each drift period because of imperfect detection of blinks and (micro)saccades." (page 5). Then, they “removed an additional 10 ms from the beginning and end of each drift period to reduce edge effects due to the filter.” We chose a different approach to “trimming” each fixation (See Fig 2). Once our velocity signals for each fixation were trimmed, we computed the mean velocity (separately for horizontal, vertical and radial signals).

### Computing medians per epoch for all measures

At this point in the analysis, each selected fixation was characterized by the following five features (one set based on horizontal signals and one based on vertical signals). All were expressed in degrees per second.

1. Slope for 50 msec segment length
2. Slope for 100 msec segment length
3. Slope for 300 msec segment length
4. Slope for 500 msec segment length
5. Mean Velocity

For each session, each random saccade task was 100,000 ms long. For each session the 100,000 ms were divided up into five 20,000 ms “epochs”. For each epoch we computed the median feature across all available fixations. If a fixation started during one of the five epochs, the fixation was assigned to that epoch.

For example, with a segment length of 300, each epoch had a median of 19 fixations. In this next step, each epoch was characterized by the median across all fixations during that epoch.

So, at this point, each epoch was characterized by the following values (one set based on horizontal signals and one based on vertical signals).

1. Median Slope for 50 ms segment length
2. Median Slope for 100 ms segment length
3. Median Slope for 300 ms segment length
4. Median Slope for 500 ms segment length
5. Median Mean Velocity

### Computing slopes of medians across epochs

**Fig 3:**
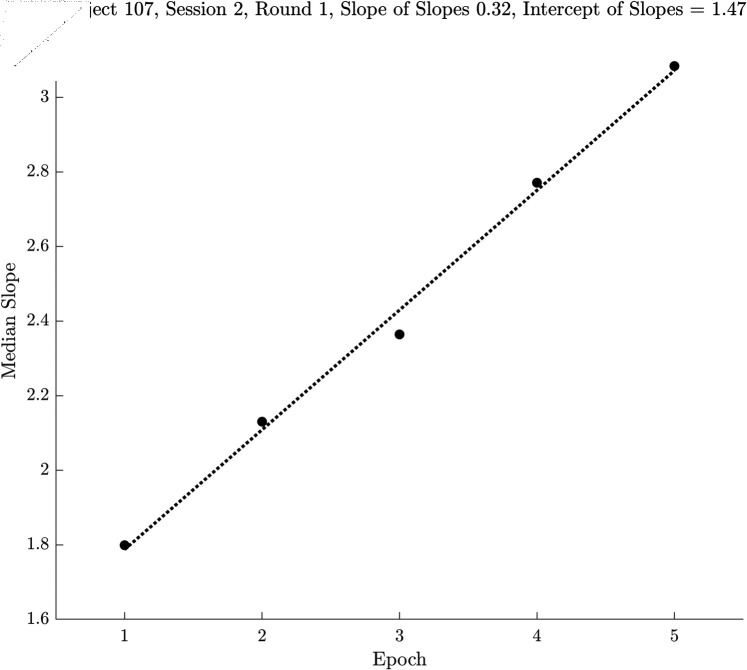
Illustration of the Slope and Intercept calculation across epochs. The black dots represent the median of slope measures (say 300 ms segment length) across fixations. The regression line was fitted to these slope estimates per epoch. After this step, each run of the random saccade task was characterized by a slope and an intercept for each of our five features for each dimension.

To understand the next step in our analysis, refer to Fig 3. Here we have five epochs of a single run of the random saccade task for a single subject. For subject 107, session two, round one the dots plotted are the median slope value across fixations in that epoch. We then computed a best fitting (in the least squares sense) regression line to the medians across epochs. This analysis provides a slope and an intercept value. In this case, the slope was 0.32 and the intercept was 1.47. When the underlying data were slopes, these new slope estimates can be thought of as “Slopes across Slopes". We also have an estimate of the “Intercepts across Slopes". If the underlying data were medians of mean velocities, these new slopes were the slopes across median velocities per epoch. So, at this point, each run of the random saccade task was characterized by a slope and intercept value for each measurement type. These slopes and intercepts were the dependent measures for the statistical analysis.

### Statistical analyses

Table 1 lists the final 20 dependent measures that were subjected to statistical analysis. The analysis employed the SAS (Cary, North Carolina) procedure PROC MIXED. It performs a linear mixed model analysis. Initially, round and session were treated as fixed repeated measures. We tested for a session main effect, a round main effect and a session-by-round interaction. In no case was there a statistically significant round main effect or session-by-round interaction. Therefore, for our final analysis we only tested for the presence of a session main effect. The distributions of our slope measures were right skewed. We therefore tested if our slope measures were statistically significantly greater than 0.0, using the Wilcoxon Signed Rank test. This test produces a *p*–*value* and a z-value statistic that represents the size of the effect. These z-values were transformed into Pearson R values using equation two [6]:

**Table 1:** Statistical Results for the session effect.

Measure	Slope Or Intercept	df (denom)	F (session)	*p*–*value* (session)	p-Corrected^b^
Hor Slope 50 ms	Slope	868	5.46	0.0197	0.0341
Hor Slope 50 ms	Intercept	800	7.00	0.0083	0.0168
Hor Slope 100 ms	Slope	867	9.19	0.0025	0.0079
Hor Slope 100 ms	Intercept	797	7.55	0.0061	0.0149
Hor Slope 300 ms	Slope	840	9.38	0.0023	0.0079
Hor Slope 300 ms	Intercept	812	15.02	0.0001	0.0022
*Hor Slope 500 ms* ^a^	*Slope*	*837*	*2.18*	*0.1403*	*0.1470*
Hor Slope 500 ms	Intercept	823	12.33	0.0005	0.0051
Hor Mean Velocity	Slope	847	4.31	0.0383	0.0468
Hor Mean Velocity	Intercept	789	5.29	0.0217	0.0341
Ver Slope 50 ms	Slope	875	11.48	0.0007	0.0051
*Ver Slope 50 ms* ^a^	*Intercept*	*813*	*0.96*	*0.3271*	*0.3271*
Ver Slope 100 ms	Slope	875	6.97	0.0084	0.0168
Ver Slope 100 ms	Intercept	809	3.91	0.0484	0.0560
Ver Slope 300 ms	Slope	877	9.80	0.0018	0.0079
Ver Slope 300 ms	Intercept	810	4.98	0.0259	0.0356
*Ver Slope 500 ms* ^a^	*Slope*	*835*	*3.20*	*0.0741*	*0.0815*
Ver Slope 500 ms	Intercept	808	5.39	0.0205	0.0341
Ver Mean Velocity	Slope	860	10.47	0.0013	0.0072
Ver Mean Velocity	Intercept	798	5.04	0.0251	0.0336
Radial Mean Velocity	Slope	861	8.77	0.0032	0.0088
Radial Mean Velocity	Intercept	795	4.82	0.0284	0.0368

^a^ Italics indicates not statistically significant session effect.

^b^ Bold text indicates p value was still statistically significant after correction for multiple comparisons.

$$R=Z/\sqrt{(}N)$$ (2)

and R can be converted into Cohen’s d as in equation three [7]:

$$d=2R/\sqrt{(}1-R^{2}).$$ (3)

*P*–*values* were corrected for multiple comparisons using the Benjamini-Hochberg approach, with alpha = 0.05.

## Results

### What are the “High Drift” fixation trajectories using the mean velocity measures?

**Fig 4:**
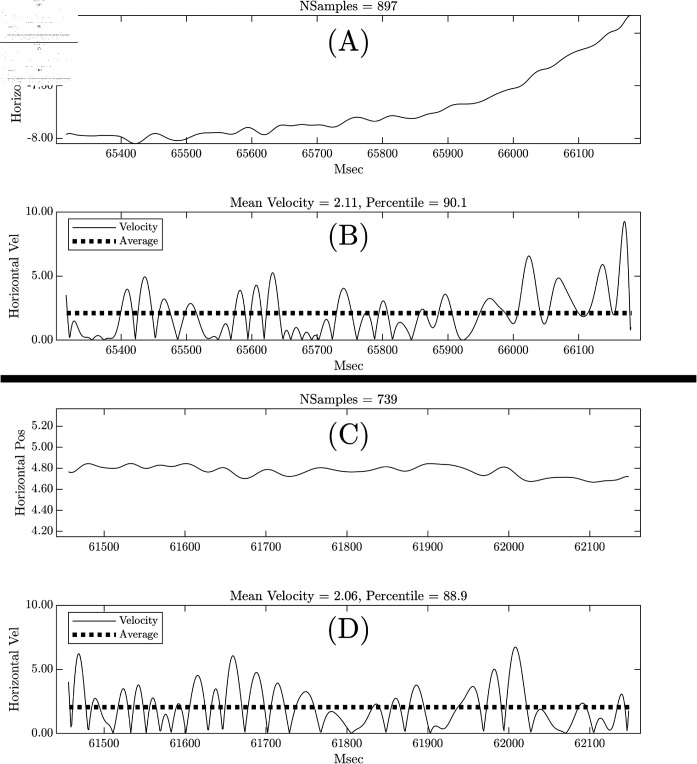
What does high mean velocity indicate about eye-position trajectories? The top panels [(A) and (B)] present data from a fixation with a horizontal mean velocity of 2.11 (90.1 percentile). The bottom two panels [(C) and (D)] present a fixation with a horizontal mean velocity that was very similar to the upper panels (mean = 2.06, 88.9 percentile). It is important to note that the y-axis range for (A) and (C) were identical. The fixation in (A) clearly has what we would term “curvilinear” drift. However, we question if the trajectory in (C) is reasonably characterized as illustrating drift in the same sense as in (A). To us, the trajectory in (C) is better characterized as irregularly oscillating.

We illustrate position signals from two fixations that were identified as having high mean velocity (Fig 4). Both the trajectory in (A) and (C) have high and similar mean velocity. The panel (A) signal unambiguously demonstrates drift. We can further specify that this is “curvilinear drift”. We are not sure in what sense the fixation trajectory in (C) illustrates the same kind of fixation drift as in (A). We think of this kind of trajectory as illustrating irregular oscillations.

The figure makes clear that high mean velocity does not detect homogeneous types of drift. We will refer to the [1] drift as “Curvilinear Drift or Irregular Oscillation".

**Fig 5:**
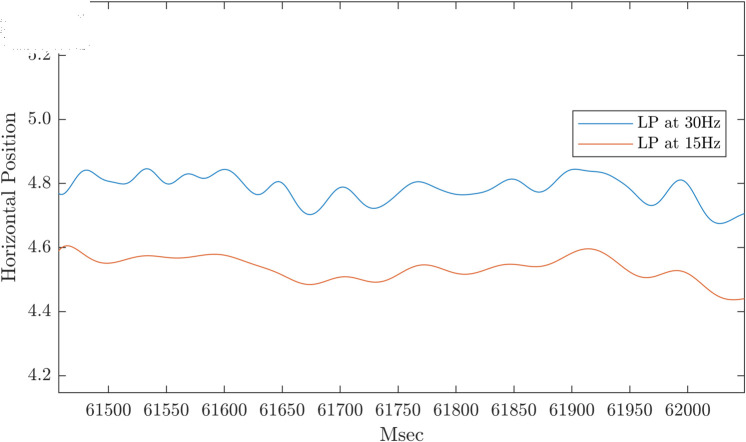
Illustration of the effect of the low pass filter cutoff on position trajectories. The upper trajectory (blue line) was a high velocity trajectory found using the mean velocity criteria. The lower trajectory (red line) was the same trajectory after prefiltering with a 15 Hz cutoff rather than a 30 Hz cutoff. Note how the trajectory was affected by the low-pass filter cutoff.

Fig 4 raises the question: to what extent were the fixation trajectories found by using mean velocity a function of the cutoff of the low-pass filter applied. In [1], the authors chose a low pass filter cutoff of 30 Hz. The rationale for choosing 30 Hz was not fully elaborated. In Fig 5, we present a fixation low-pass filtered at 30 Hz (blue line) and low-pass filtered at 15 Hz (red line). It was evident that the type of fixation trajectory found by using mean velocity as a measure of drift depends on the low-pass filter cutoff. The basis for a low-pass filter cutoff needs further justification, in our view.

### Test of the session effect

Table 1 presents the results of the statistical tests of the session effect. The variables printed in italics were not statistically significant. The *p*–*values* that were shown in bold font were still statistically significant after correction for multiple comparisons, using the Benjamini-Hochberg (false-discovery rate, FDR) procedure with a specified FDR level set to 0.05. Nineteen of 22 tests of session effects were statistically significant after correction for multiple comparisons.

### Are the slope measures greater than 0.0?

Below, we ask the question: Are the slope estimates greater than 0.0. We employed the Wilcoxon Signed Rank tests and the results are shown in Table 2. Only data from round one were included in this analysis. This test produces a z-value statistic. This can be converted into a Pearson R which can then be converted into a Cohen’s d (see methods). The effect sizes were generally in the moderate to large range. All tests were still statistically significant after correction for multiple comparison using the Benjamini-Hochberg approach. The largest effect size was for mean radial velocity.

**Table 2:** Results of Wilcoxon signed rank tests: is slope or mean velocity greater than 0.0?

	Horizontal (P-value Corrected)			Vertical (P-value Corrected)		
	Pearson	Cohen’s	P-value	Pearson	Cohen’s	P-value
Slope (50 msec)	0.28	0.58	2.95E-12	0.34	0.73	1.68E-17
Slope (100 msec)	0.26	0.54	7.56E-11	0.34	0.72	2.48E-17
Slope (300 msec)	0.2	0.41	4.08E-07	0.21	0.42	1.91E-07
Slope (500 msec)	0.2	0.4	6.43E-07	0.23	0.48	4.47E-09
Mean Velocity - Filtered	0.28	0.59	1.75E-12	0.35	0.75	2.57E-18
Mean Radial Velocity	0.37	0.8	1.36E-34			

### Plots of slope and intercept effects

**Fig 6:**
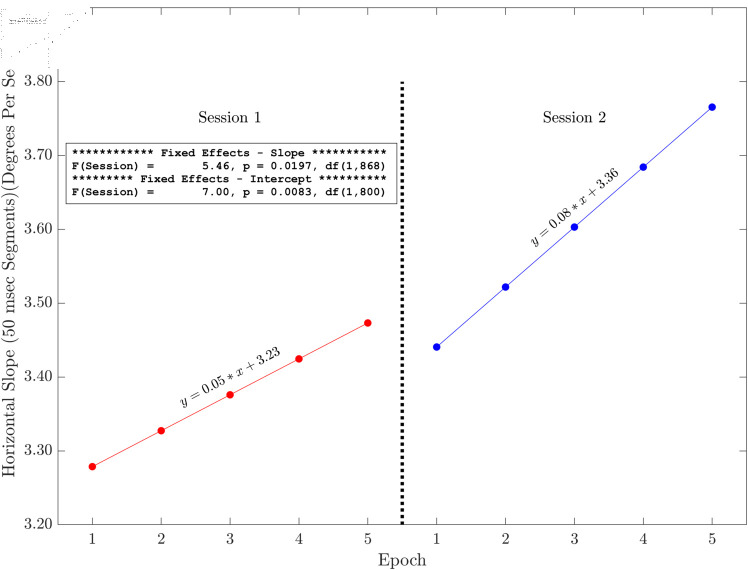
Illustration of the effects of slope and intercept for horizontal linear drift with a 50 ms segment length. There were statistically significant session effects on the slope and the intercept. Both the slope and the intercept increase in session two. The session two data start off (epoch one) close to where the session one data (epoch five) end.

**Fig 7:**
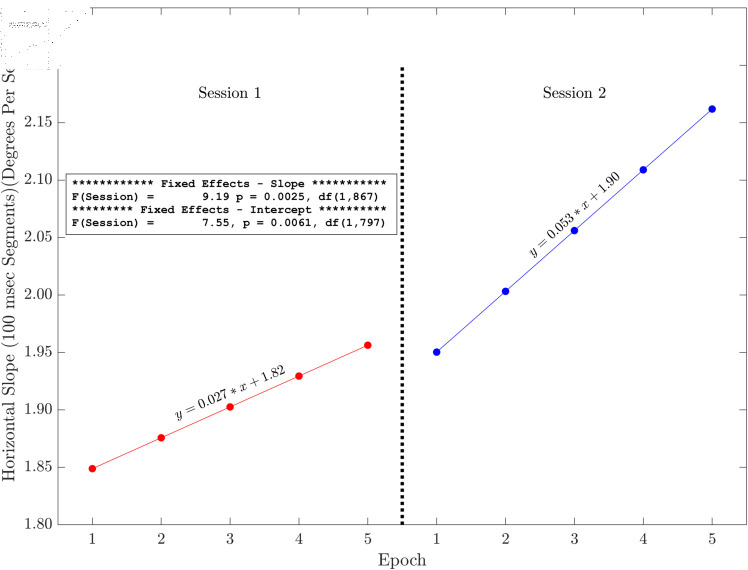
Illustration of the effects of slope and intercept for horizontal linear drift with a 100 ms segment length. There were statistically significant session effects on the slope and the intercept. Both the slope and the intercept increase in session two. The session two data start off (epoch one) close to where the session one data (epoch five) end.

**Fig 8:**
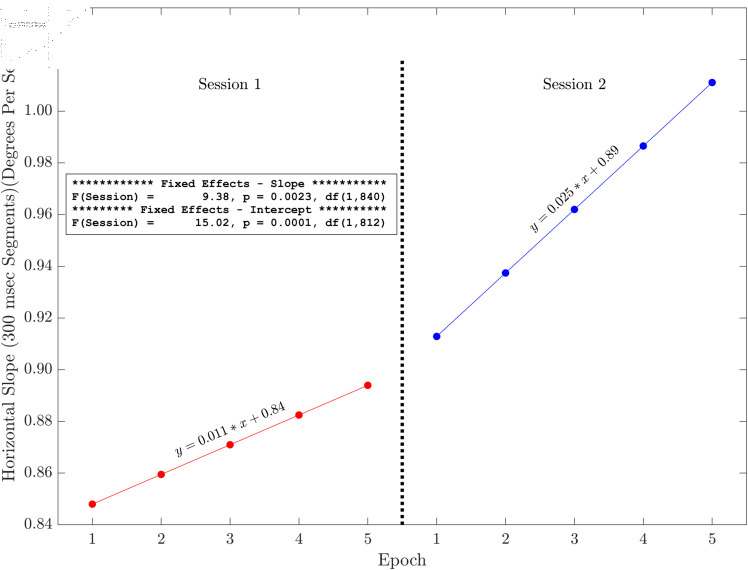
Illustration of the effects of slope and intercept for horizontal linear drift with a 300 ms segment length. There were statistically significant session effects on the slope and the intercept. Both the slope and the intercept increase in session two. The session two data start off (epoch one) close to where the session one data (epoch five) end.

**Fig 9:**
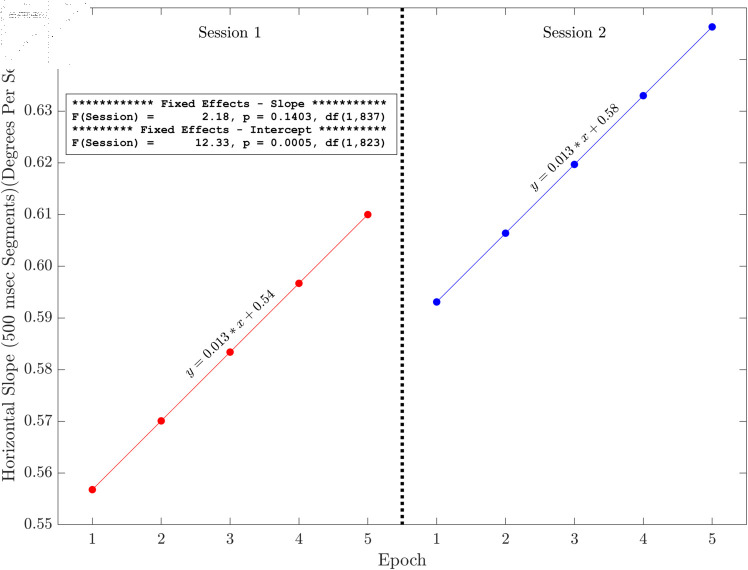
Illustration of the effects of slope and intercept for horizontal linear drift with a 500 ms segment length. There was a statistically significant session effect on intercept but not on slope. The intercept increases in session two. The session two data start off (epoch one) close to where the session one data (epoch four) end.

**Fig 10:**
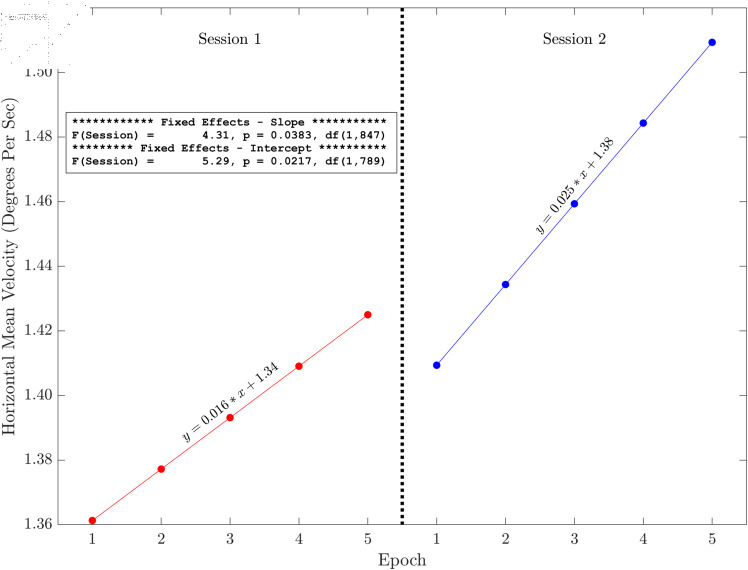
Illustration of the effects of slope and intercept for horizontal mean velocity. There were statistically significant session effects on the slope and the intercept. Both the slope and the intercept increase in session two. The session two data start off (epoch one) close to where the session one data (epoch four) end.

**Fig 11:**
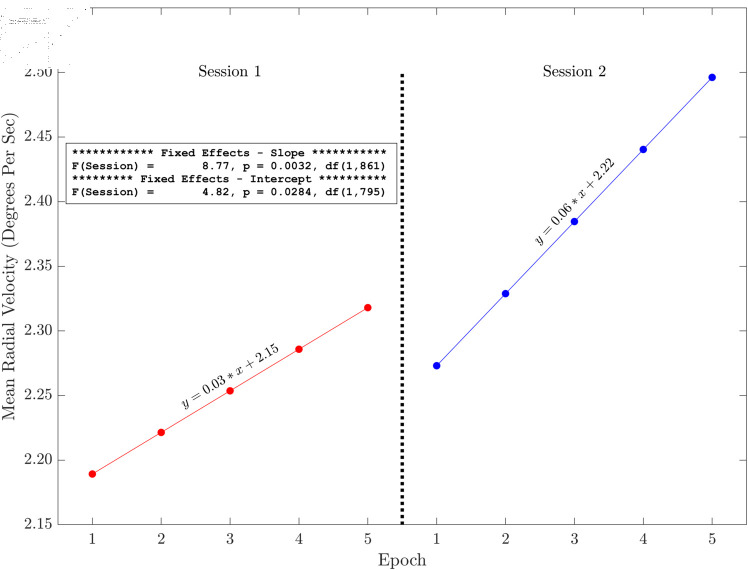
Illustration of the effects of slope and intercept for mean radial velocity. There were statistically significant session effects on the slope and the intercept. Both the slope and the intercept increase in session two. The session two data start off (epoch one) close to where the session one data (epoch four) end.

Here we present the results of the tests of the slopes and intercepts estimated for each session across the five methods for horizontal and radial signals only (see Figs 6, 7, 8, 9, 10 and 11). The same figures for the vertical signals can be found at: https://hdl.handle.net/10877/20418/PlotsOfSlopeAndInterceptEffects_VerticalPosition.zip. Of the 11 tests of the session effect, nine were statistically significant. The only exceptions were the absence of a session effect for the horizontal and vertical slope with a 500 ms segment length. The slopes were all positive. The intercepts were all positive, and the intercept for session two was higher than the intercept for session one. For segments lengths 50, 100 and 300 ms, the session two data pick up where the session one data end. For the 500 ms segment length, the horizontal mean velocity, and the radial mean velocity the session two data begin at a value closer to the session one, epoch four level. The results for the vertical positions were generally very similar.

### Correlations between linear drift and mean velocity drift

Table 3 presents the Spearman inter-correlations between the linear drift methods and the mean velocity method. The correlations were substantial and were somewhat higher for shorter linear drift segments (50 and 100 ms) than for longer linear drift segments (300 and 500 ms). The median correlation was 0.48. We also correlated the eight linear drift slope measures with the mean radial velocity measures (Spearman r Range = 0.29 to 0.49, median = 0.41).

**Table 3:** Table of correlations between linear drift measures and mean velocity drift.

Linear	Horizontal	Vertical
Drift Segment Length	Mean Velocity	Mean Velocity
50 ms	0.47	0.60
100 ms	0.48	0.59
300 ms	0.43	0.55
500 ms	0.32	0.46

## Discussion

The main finding of this study is that, based on multiple measures, there was fixation drift over time in our 100 second random saccade task. In all of our several measures of drift, there was strong statistical evidence that the slope estimates were greater than zero (Table 2). Effect sizes were in the moderate to large range. Recall that, on each visit, subjects performed the random saccade task in two sessions separated by approximately 20 minutes. In many cases (19 of 22 measures) there were statistically significant effects of session on either the slope or the intercept or both of the drift regression line computed for each run of the task (see Fig 3 and Table 1). In 10 of our 11 tests of the effect of session on slope estimates, the slope of the second session was greater than that from the first session. Similarly, for nine of our 10 tests of the session effect on the intercept of the drift regression line, the intercept was larger for session two than for session 1. Often, the session two drift level for epoch one began near the final drift (epoch five) estimate for the first session. This was true for Figs 6, 7 and 8. For Figs 9, 10 and 11 the estimate of drift for the first epoch of session two was more similar to the drift level for the 4 epoch of session one rather than the 5. The same was true for three of the five analyses of drift in the vertical position channel (data available at: https://hdl.handle.net/10877/20418/PlotsOfSlopeAndInterceptEffects_VerticalPosition.zip). In summary, our evidence is that drift increases within each run of the random saccade task and that session two data have increased slope of drift estimates compared to session one. Also, generally, the drift estimates for session two began where the estimates for session one left off.

Our drift measures were of two types: linear drift based on slope estimates per fixation and curvilinear or irregularly oscillating “drift” assessed with mean velocity of the filtered eye-position signals. This later measure was employed by an earlier paper on fixation drift over time on task [1]. For us, it was not intuitive what sort of drift would be detected with the mean velocity approach, so we investigated. Fig 4 illustrates two fixation trajectories ((A) and (C)) that have very similar mean velocity but have markedly different trajectories. In one case (Fig 4(A)) we have a clear case of curvilinear drift over time. However, in another case (Fig 4(c)) we have a pattern of irregular oscillation. The linear drift measures were substantially correlated with the mean velocity drift measures. (See Table 3. Correlations ranged from 0.32 to 0.60, with a median of 0.48.)

The presence of irregular oscillation suggests that the “drift” trajectories uncovered using the mean velocity of filtered signals might be affected by the cutoff of the low-pass filter applied to the position data. We demonstrated this effect in Fig 5. In our view, a detailed justification for the selection of the low-pass filter cutoff is required prior to further use of mean velocity as a measure of drift.

Di Stasi, *et al*. [1] evaluated fixation drift over two hours whereas we demonstrate fixation drift over 100 seconds. The Di Stasi, *et al*. [1] study evaluated drift while subjects performed a complex task (air traffic controller game). So, the present study extends the findings of increased drift to a much shorter and much simpler task.

We cannot provide a detailed theory regarding the cause of the fixation drift that we noted in our 100 sec saccade tracking task. It is simply beyond the scope of this investigation. Keeping the eye focused on a small visual dot in space might require mechanical factors (oculomotor muscles) which may become fatigued, neural mechanisms which may be depleted, or psychological factors such as increasing apathy and boredom. The extraocular muscles are known to be fatigue resistant [8], but this is not the same as saying that they are indefatigable. According to Krauzlis *et al*. [9]:

Eye position during fixation is actively controlled and depends on bilateral activity in the superior colliculi and medio-posterior cerebellum; disruption of activity in these circuits causes systematic deviations in eye position during both fixation and smooth pursuit eye movements.

Perhaps fatigue is such a disruption. The locus coeruleus is a small, bluish-grey nucleus in the brainstem that is the brain’s primary source of norepinephrine (NE). According to Ghosh and Maunsell [10]:

LC-NE neurons spike in response to a visual stimulus appearing in the contralateral hemifield only when that stimulus is attended. This spiking is associated with enhanced behavioral sensitivity, is independent of motor control, and is absent on error trials.

Perhaps some depletion of NE or neuronal fatigue in this region is associated with increasing fixation drift. A number of large-scale neuronal networks are proposed to be involved in visual attention [11]. Any reduction in the efficacy of these networks could be associated with increased fixation drift. See [12–14] for discussions of the relationship between boredom and eye-movements.

### Future directions

An interesting future study would be to run our task for five to 10 minutes to evaluate drift changes over a much longer continuous interval. Another interesting future study would be to evaluate drift over two consecutive runs of the random saccade task, with variation in time between the first and the second task. This would allow us to understand how much time between tasks is required to make the session two data look like the session one data. This would tell us how much rest time it takes to remove any carry-over fatigue effects from one run to the next.

## Conclusion

Our results, taken together, provide evidence of increased fixation drift over time within each run of our 100 sec random saccade task. The second run of the task, approximately 20 min later, typically has drift that picks up where the session one drift estimates end, and typically, the increase in drift from epoch to epoch was greater in session two than in session one. Also, drift trajectories using mean velocity to assess drift often demonstrate curvilinear drift or irregular oscillation.
